# Piperlongumine Inhibits Akt Phosphorylation to Reverse Resistance to Cisplatin in Human Non-Small Cell Lung Cancer Cells *via* ROS Regulation

**DOI:** 10.3389/fphar.2019.01178

**Published:** 2019-10-11

**Authors:** Chao Zhang, Lian-Jun He, Yi-Bao Zhu, Qing-Zhu Fan, Dong-Dong Miao, Sheng-Peng Zhang, Wen-Ying Zhao, Xiao-Ping Liu

**Affiliations:** ^1^Center of Drug Screening and Evaluation, Wannan Medical College, Wuhu, China; ^2^Anhui Provincial Engineering Research Center for Polysaccharide Drugs, Wannan Medical College, Wuhu, China; ^3^Oncology Department, The First Affiliated Hospital of Wannan Medical College, Wuhu, China

**Keywords:** Akt, multidrug resistance, piperlongumine, NSCLC, apoptosis

## Abstract

Resistance is a major concern when administering chemotherapy to patients with non-small cell lung cancer (NSCLC). Chemosensitizer are agents that can reverse resistance to chemotherapeutic drugs, thereby enhancing the chemosensitivity of tumor cells. Thus, their development will improve therapeutic efficacy in cancer. However, few effective chemosensitizer have been identified to date. Piperlongumine (PL) has been shown to effectively reverse resistance to chemotherapeutic drugs in several types of cancers. However, the mechanisms associated with the chemotherapy resistance reversal effect of PL and its regulation of target factors in chemotherapy resistance cells are still unclear. This study investigated the reversal effect of PL both *in vitro* and *in vivo*, and provided evidence that PL inhibited the phosphorylation of Akt *via* the accumulation of reactive oxygen species in chemotherapy resistance cells. Consequently, various Akt activation-dependent genes caused a reduction of drug efflux and induction of apoptosis in cisplatin-resistant A549 NSCLC cells. Our results indicate that Akt phosphorylation may play a functional role in the reversal effect of PL and contribute, at least in part, to the treatment outcomes of patients with chemotherapy resistance.

## Introduction

Most cancer-related death is caused by non-small cell lung cancer (NSCLC) in China (Bray et al., 2018). Chemotherapy is the main clinical therapy for NSCLC (Hirsch et al., 2017; Herbst et al., 2018), but the rapid development of chemoresistance is a major obstacle to the successful treatment of NSCLC (Jiang et al., 2018; Kwon et al., 2018). The identification of effective chemosensitizer that are able to reverse chemotherapy resistance and work with anticancer drugs is a promising strategy for increasing the success rate of chemotherapy (Fennell et al., 2016). However, most of chemosensitizers displayed unacceptable levels of toxicity even if administered at effective doses in clinical trials (Kim et al., 1993; Ferry et al., 1996; Thomas and Coley, 2003), leading to restriction of their application in clinical settings. Thus, identification of novel chemosensitizers with higher efficacy and lower toxicity is an essential task.

Piperlongumine (PL) is a natural product obtained from the fruit of long pepper and a form of traditional Chinese medicine (Zou et al., 2016; Hang et al., 2018) that exhibits a wide range of pharmacological properties (Adams et al., 2012; Wang et al., 2013; Han et al., 2014; Prasad and Tyagi, 2016) and has also been shown to reverse chemotherapy resistance in various cancers (Roh et al., 2014; Kang and Yan, 2015; Patel et al., 2015; Wang et al., 2015). However, the mechanisms underlying its effects are unclear.

In this study, key targets and mechanisms of the reversal effect of PL were investigated *in vitro* and *in vivo*. The results indicated that reactive oxygen species (ROS) suppressed phosphorylation of Akt may play a critical role in the chemotherapy resistance reversal effect of PL by reducing drug efflux and promoting the apoptosis of NSCLC cells. Together with previously published data, our results imply that PL may have prospect of clinic treatments employed in NSCLC patients with chemotherapy resistance.

## Materials and Methods

### Materials

PL was purchased from Selleck Chemicals (Houston, TX, USA; purity: 99.33%); dimethyl sulfoxide (DMSO), 3-[4,5-dimethyl-2-thiazol]-2,5-diphenyltetrazolium bromide (MTT), and N-Acetyl-L-cysteine (NAC) were purchased from Sigma-Aldrich (Darmstadt, Germany); cisplatin (Cis) was from Hansoh Pharmaceutical Group (Lianyungang, Jiangsu, China); rabbit polyclonal anti-Akt, anti-phosphorylated Akt (p-Akt, Ser-473), anti-p-forkhead box O3 (p-FoxO3a, Ser-318/Ser-321), anti-p-mTOR (p-mTOR, Ser-2448), anti-p-PTEN (p-PTEN, Ser-280) and rabbit monoclonal anti-p-Bcl-2-associated death promoter (p-BAD; Ser-136 and Ser-112), anti-mTOR, and anti-PTEN antibodies were from Cell Signaling Technology (9272, 4060, 9465, 2971, 9551, 2983, 9559, 5286, and 5284, respectively; Danvers, MA, USA). Rabbit monoclonal anti-FoxO3a, anti-nuclear factor erythroid 2-related factor 2 (NRF2), anti-BAD, anti-Bcl-extra large (anti-Bcl-xL), and mouse monoclonal anti-GAPDH antibodies were purchased from Abcam (ab53287, ab62352, ab32445, ab32370, and ab8245, respectively; Cambridge, MA, USA). Mouse monoclonal anti-p53 and anti-P-glycoprotein (P-gp) antibodies were purchased from Santa Cruz Biotechnology (sc-47698 and sc-55510, respectively; Dallas, TX, USA). Hoechst 33258 staining buffer, ROS assay kit, and RIPA lysis buffer were purchased from Beyotime Institute of Biotechnology (C1017, S0033 and P0013B, respectively; Haimen, Jiangsu, China). In Situ Cell Death Detection Kit (POD) was purchased from Roche (Mannheim, Germany; cat. no. 11684817910). FITC-Annexin V Apoptosis Detection Kit I was purchased from BD Biosciences (San Jose, CA, USA; cat. no. 556547). The Protease and Phosphatase Inhibitor Cocktail, Rhodamine 123 (Rh-123), BCA Protein Assay Kit, and EdU Imaging Kit were purchased from Thermo Fisher Scientific (78440, R302, 23225 and C10337, respectively; Waltham, MA, USA). The Western Blot System was purchased from ProteinSimple (SM-W004, DM-001 and DM-002, respectively; San Jose, CA, USA).

### Cell Culture

The human NSCLC cell line A549 was purchased from Type Culture Collection of the Chinese Academy of Sciences (Shanghai, China); Cisplatin resistance cell line A549/Cis was purchased from China Infrastructure of Cell Line Resource (Beijing, China). The two cell lines were grown in F12K medium supplemented with 10% (v/v) fetal bovine serum (FBS; Thermo Fisher Scientific, Inc., Waltham, MA, USA) and maintained in a humidified atmosphere with 5% CO_2_ at 37˚C.

### Cell Viability Assay

The effects of drug treatments on cell viability were quantified using the MTT assay. Five thousand cells per well were seeded in 96-well plates overnight and then treated with the drugs (PL: 100 μM, 2-fold dilution for eight concentrations; Cis: 100 μg/ml, 2-fold dilution for 10 concentrations). Complete F12K medium without drug was added to the blank wells. Control cells were treated with DMSO only. After incubation for 24 or 48 h, 10 μl MTT was added to each well and cells were incubated at 37°C for 4 h. Then, the medium was removed and 150 μl DMSO was added, followed by gentle shaking. The optical density (OD) of the released color was read at 570 nm and the cell growth inhibition rate was calculated using the following formula: growth inhibition rate = (OD value of control - OD value of test)/(OD value of control - OD value of blank) x 100%.

### Chemotherapy Resistance Reversal Assay

A concentration of 5 μM PL, which was lower than the IC_10_ value obtained following the MTT assay of PL alone in A549/Cis cells, was designated as the reversal concentration of PL in the following experiments.

A549/Cis cells were seeded in 96-well plates overnight in complete F12K medium and pretreated with 5 μM PL for 4 h, followed by treatment with serial dilutions of Cis for 24 h (100 μg/ml as the starting concentration, twofold dilution for 10 concentrations). Control cells were treated with Cis alone. The IC_50_ of each treatment was calculated from the MTT assay. The reversal fold (RF) was defined as: IC_50_ value of Cis alone/IC_50_ value of Cis combined with PL. Cells were also pretreated with 3 mM NAC for 2 h prior to PL exposure, followed by analysis of NAC’s effect on the reversal effect of PL.

### Apoptosis Assay

A549/Cis cells were divided into the following four treatment groups: DMSO (control), 5 μM PL, 37.5 μg/ml Cis, and a combination of 5 μM PL and 37.5 μg/ml Cis. Cells were plated in 6-well plates at an initial density of 2.4 × 10^5^ cells/well for 8 h in complete F12K media. Cells were starved in serum-free F12K medium for 16 h, followed by treatment for 8 h. Cells were harvested, washed twice with phosphate-buffered saline (PBS, Thermo Fisher Scientific), evaluated for apoptosis using the FITC-Annexin V Apoptosis Detection Kit I (BD Biosciences, San Jose, CA, USA), and analyzed using the Novocyte flow cytometer (ACEA Biosciences, San Diego, CA, USA) with NovoExpress 1.2.4 software. For NAC blocking experiments, cells were divided into the following six treatment groups: DMSO (control), 5 μM PL, 37.5 μg/ml Cis, a combination of 5 μM PL and 37.5 μg/ml Cis, a combination of 5 μM PL and 3 mM NAC, and a combination of 5 μM PL, 3 mM NAC, and 37.5 μg/ml Cis. The treatment process was the same as described above, except that groups with NAC treatment were pretreated with 3 mM NAC for 2 h after starvation in serum-free medium for 14 h.

### Intracellular Accumulation of Rhodamine-123

A549 and A549/Cis cells were plated in 6-well plates and allowed to attach overnight in F12K media. Then, A549/Cis cells were cultured for 24 h in the absence or presence of 5 and 10 μM PL, while A549 cells were treated with DMSO for 24 h, harvested, resuspended in at 1 × 10^6^ cells/ml Hank’s Balanced Salt Solution (HBSS, Thermo Fisher Scientific) with 5 μg/ml Rh-123, and incubated at 37°C, 5% CO_2_ for 30 min. Mean fluorescence intensity of intracellular Rh-123 was detected using flow cytometry at excitation and emission wavelengths of 488 nm and 525 nm, respectively.

### Western Blot Analysis

A549/Cis cells were cultured as described above in T-25 flasks. After treatment with 5 μM PL for 24 h, cells were collected, centrifuged, and washed with 1x PBS. Whole cells or tumor tissues were lysed in RIPA lysis buffer with 1x Protease and Phosphatase Inhibitor Cocktail. Protein quantification was conducted using the BCA Protein Assay Kit. Western blotting using ProteinSimple’s WES system was performed according to the manufacturer’s instructions. Briefly, 5 μl of 0.4 μg/μl total protein lysate was loaded onto a 12–230 or 66–440 kDa Wes assay plate (ProteinSimple, San Jose, CA) where 400 nl sample was withdrawn through a capillary, subjected to electrophoretic separation of proteins by size and followed by HRP-based detection of proteins of interest using an HRP-conjugated secondary antibody and the following primary antibodies: anti-Akt, anti-FoxO3a, anti-Nrf2, anti-P-gp, anti-p53, anti-BAD, anti-mTOR, anti-PTEN, anti-GAPDH, and anti-BCL-xL were 1:50 dilution, while anti-phospho Akt (Ser473), anti-phospho FoxO3a (Ser318/321), anti-phospho mTOR (Ser2448), anti-phospho PTEN (Ser380), anti-phospho BAD (Ser112), and anti-phospho BAD (Ser136) were 1:25 dilution. To facilitate data analysis, phospho-proteins were normalized to corresponding proteins, respectively. Nrf2, P-gp, p53, BCL-xL were normalized to loading control (GAPDH). The quantitative analysis was performed using ImageJ software (version 1.8; National Institutes of Health, Bethesda, MD, USA).

### Measurement of Reactive Oxygen Species Generation

Cellular ROS content was measured by flow cytometry and fluorescence microscopy for quantitative and qualitative evaluation. A549/Cis cells were plated in 6-well plates at a density of 2.4 × 10^5^ cells/well and allowed to attach overnight, and then exposed to 5 or 10 μM PL for 4 h (for positive control, cells were exposed to 50 μg/ml Rosup for 0.5 h). Cells were stained with 10 μM DCFH-DA (Beyotime Biotech, Nantong, China) at 37°C for 30 min, and then washed three times in serum-free medium. For flow cytometry, cells were collected and fluorescence was analyzed at excitation and emission wavelengths of 488 nm and 525 nm, respectively. For fluorescence microscopy, cells were collected and photos were obtained using the EVOS FL Imaging System (Thermo Fisher Scientific). In the same experiments, cells were pretreated with 3 mM NAC for 2 h prior to PL exposure and analysis of the ROS generation blocking effect of NAC. Quantitative analysis was performed using ImageJ software (version 1.8; NIH).

### Cell Proliferation Assay

A549/Cis cells were plated in 12-well plates at 1.0 × 10^4^ cells/well and allowed to attach overnight. Then, cells were starved in serum-free medium for 16 h, followed by treatment for 24 h with DMSO, Cis (10, 20, and 40 μg/ml), Cis (10 and 20 μg/ml) plus 5 μM PL, and Cis (10, 20, and 40 μg/ml) plus 5 μM PL and 3 mM NAC. Cell proliferation was analyzed using the Click-iT™ Plus EdU Alexa Fluor^™^ 488 Imaging Kit. Briefly, cells were incubated with 1x EdU solution for 2 h and fixed in 3.7% formaldehyde. The cells were permeabilized in 0.5% Triton X-100 and incubated with EdU reaction cocktail. The nucleus was stained with Antifade Mountant with DAPI (Thermo Fisher Scientific). Photos were obtained using the EVOS FL Imaging System. Quantitative analysis was performed using ImageJ (version 1.8; NIH).

### *In Vivo* Antitumor Study

All animal study procedures complied with the Wannan Medical College’s Policy on the Care and Use of Laboratory Animals. All experiments were performed in accordance with the protocols approved by the Wannan Medical College Animal Policy and Welfare Committee. Six-week-old athymic BALB/c nude mice (equal number of males and females) were purchased from Cavens Lab Animal Inc. (Changzhou, Jiangsu, China). A549/Cis cells (2 × 10^6^) were injected subcutaneously into the flank. Treatment began when tumors reached a volume of 60–70 mm^3^ (= day 0). Mice were randomized into four treatment groups: vehicle, PL, Cis, and PL plus Cis. Mice were treated by intraperitoneal (i.p.) injection of 2.5 mg/kg PL once per day, by i.p. injection of 5 mg/kg Cis once per week, or with a combination of PL and Cis according to the same schedule. Tumor volume and body weight were measured three times per week. The mice were sacrificed on day 21 and tumors were isolated, weighed, and analyzed by immunoblotting and the In Situ Cell Death Detection Kit (POD). Quantitative analysis was performed using ImageJ. For histological evaluation, normal tissues from vital organs and tumors were isolated, fixed in formalin, paraffin-embedded, sectioned, and stained with hematoxylin and eosin (H&E).

### Statistical Analysis

Results are presented as the mean ± standard deviation (SD) of at least three independent experiments for each group. Statistical differences were determined by analysis of variance with Holm’s post-hoc test for multiple comparisons or two sample *t*-tests for independent samples using SPSS 19.0 software (IBM Corp., Armonk, IL, USA). All graphs were prepared using GraphPad Prism 5.0 (GraphPad; San Diego, CA, USA). *P* < 0.05 was considered statistically significant.

## Results

### PL Enhances the Chemosensitivity of A549/Cis Cells to Cis

**Figure 1:**
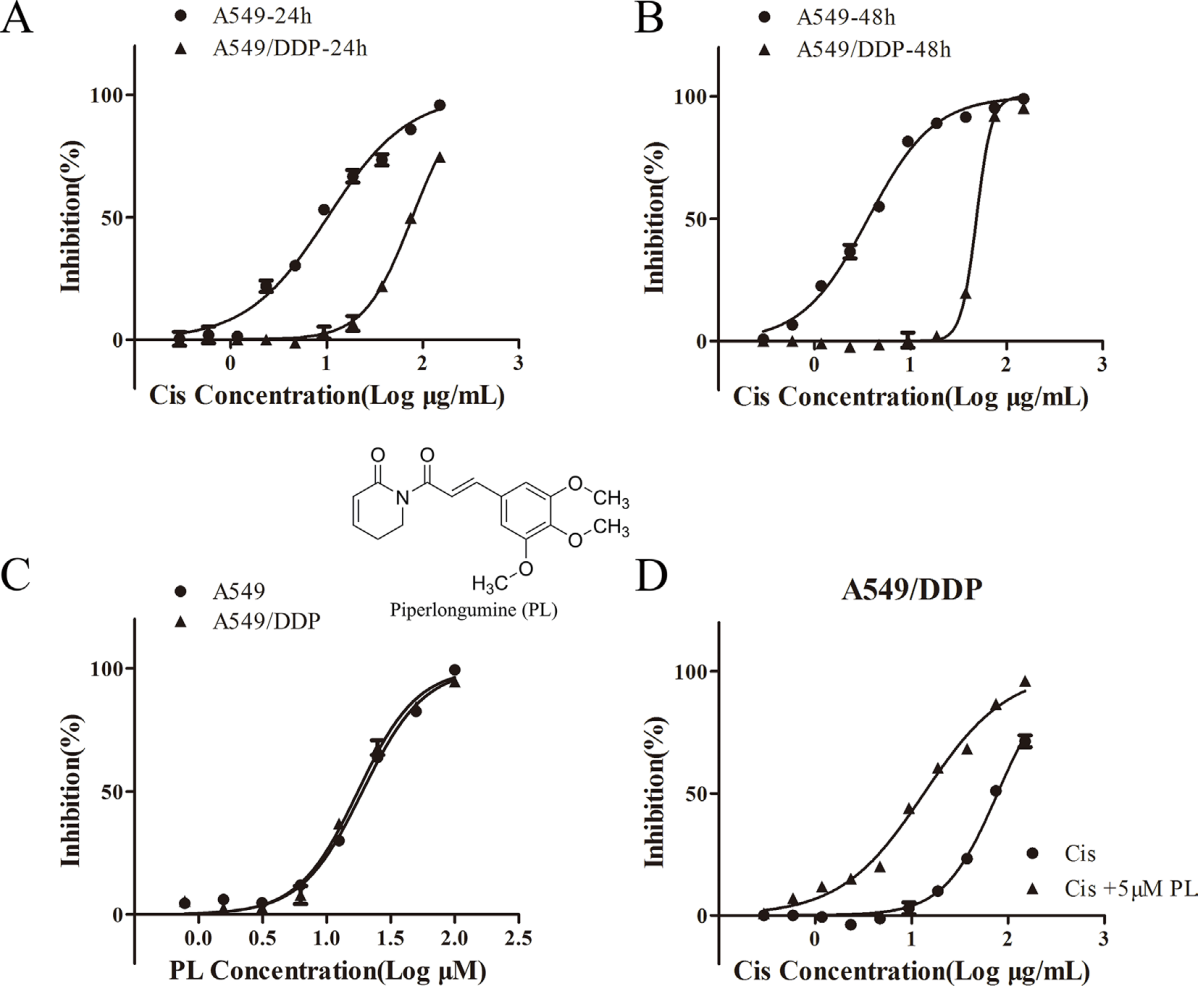
Piperlongumine (PL) enhances the chemosensitivity of A549/Cis cells to cisplatin. A549 and A549/Cis cells were treated with various concentrations of cisplatin at 24 h **(A)** or 48 h **(B)**, or treated with various concentrations of PL at 24 h **(C)**. **(D)** A549/Cis cells were treated with cisplatin alone or in combination with PL at 24 h and the cell viability was measured by an MTT assay. DMSO was used as the negative control. Five replicates were made for each concentration of the drugs. The results are expressed as the mean ± SD (n = 3) of three independent experiments.

To validate the resistance to Cis in A549/Cis cells, the cytotoxicity of Cis in parental A549 cells and resistant A549/Cis cells was examined by the MTT assay. In A549/Cis cells, the Cis IC_50_ value was 7.63-fold higher than in parental A549 cells at 24 h (**Figure 1A**) and was 13.44-fold higher at 48 h (**Figure 1B**). The intrinsic *in vitro* toxicity of PL on A549 and A549/Cis cells was also evaluated using the MTT assay. PL inhibited the growth of both cell types in a dose-dependent manner *in vitro*. The IC_50_ values of PL for A549 and A549/Cis cells were comparable, indicating that A549/Cis cells were not cross-resistant to PL (**Figure 1C**). The IC_10_ value of PL for A549/Cis cells at 24 h was ∼10 μM, so 5 μM of PL was used in subsequent experiments. For A549/Cis cells, the IC_50_ values of Cis alone and Cis combined with 5 μM PL at 24 h were 78.01 ± 0.2764 μg/ml and 13.45 ± 0.1347 μg/ml, respectively (**Figure 1D**). The reversal effects of 5 μM PL for A549/Cis cells were 5.8-fold. These results demonstrated that PL significantly increased the sensitivity of A549/Cis cells to Cis.

### PL Treatment Combined With Cis Induces Apoptosis in A549/Cis Cells

**Figure 2:**
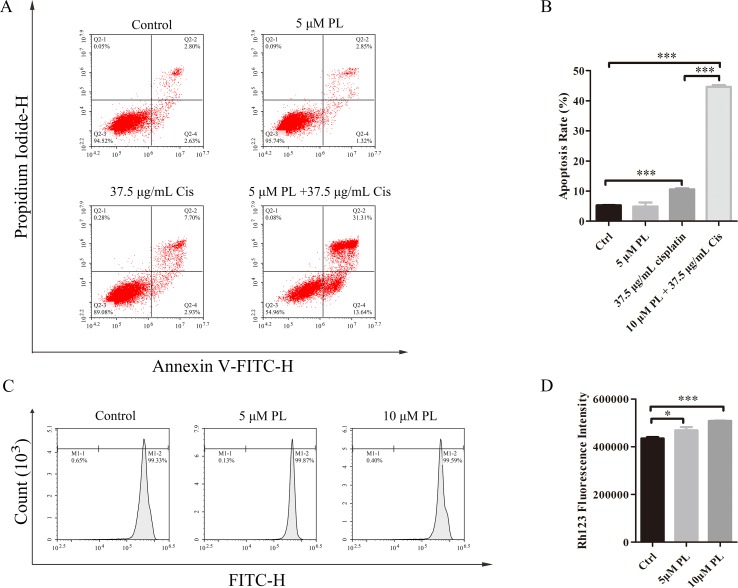
Piperlongumine (PL) reverses the resistance of A549/Cis cells by promoting apoptosis and reducing cisplatin efflux. **(A)** PL combined with cisplatin induced cell apoptosis in A549/Cis cells. A549/Cis cells were treated with DMSO, 5 μM PL, 37.5 μg/ml cisplatin or 5 μm PL, and 37.5 μg/ml cisplatin combined for 24 h. **(B)** the percentage of apoptosis cells were determined by Annexin–V/PI staining and flow cytometry in three independent experiments and graphed by GraphPad Prism 5. PI, propidium iodide. **(C)** PL reduced the intracellular accumulation of Rh-123 in A549/Cis cells. A549/Cis cells were treated with DMSO (control), 5 μm PL, or 10 μM PL. After 24 h, cells were incubated with 5 μg/ml Rh-123 for 30 minutes. Intracellular fluorescence was measured using flow cytometry to assess the function of P-gp. **(D)** A graph representing the analysis of intracellular Rh-123 fluorescence. Data presented are mean ± SD values from at least three independent experiments. Bars = SD. *P < 0.05, ***P < 0.005.

One mechanism associated with the reversal effect of chemosensitizers in resistance cells is they can induce apoptosis of these cells (Chapman et al., 1994; Chapman et al., 1995; Gottesman et al., 2002; Fernald and Kurokawa, 2013). To investigate whether PL reverses the resistance of A549/Cis cells by promoting apoptosis, flow cytometry was used to evaluate the apoptosis rates in untreated A549/Cis cells and cells treated with PL, Cis, or PL combined with Cis. Only a few cells underwent apoptosis after treatment with 5 μM PL (**Figures 2A, B**); however, with 37.5 μg/ml Cis treatment, the apoptosis rate increased. The combined treatment of 5 μM PL with 37.5 μg/ml Cis significantly increased the rate of apoptosis compared with Cis alone in A549/Cis cells. These results showed the stronger effects of PL combined with Cis in inducing A549/Cis cell apoptosis.

### PL Treatment Inhibits Drug Efflux From A549/Cis Cells

**Figure 3:**
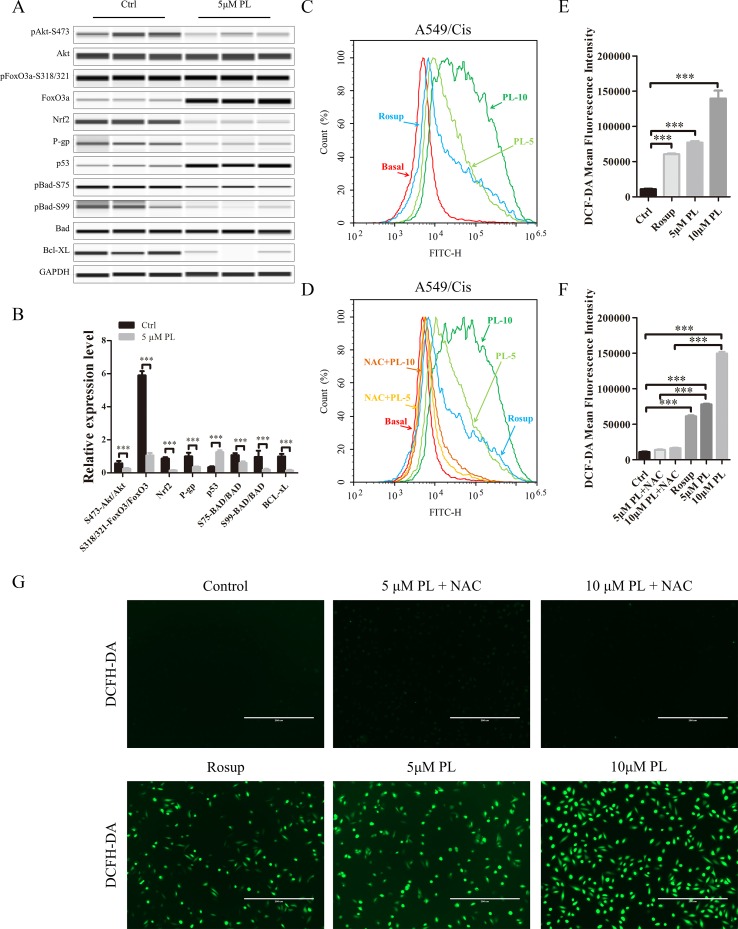
Intracellular ROS generation induced by PL was blocked by NAC. **(A)** PL downregulates the network of Akt signaling to reverse resistance of A549/Cis cells. A549/Cis cells were treated with DMSO or 5 μM PL for 24 h. The effects on Akt, Akt (Ser473), FoxO3a, FoxO3a (Ser318/321), Nrf2, P-gp, p53, BAD, BAD (Ser75), BAD (Ser99), and Bcl-xL protein expression were evaluated by western blot analysis. **(B)** relative protein expression levels were quantified using ImageJ. Phospho-protein levels were normalized to corresponding protein and the other protein levels were normalized to GAPDH. Data are expressed as the mean ± SD of three independent experiments. **(C**, **D**, **E)** intracellular ROS generation induced by increasing doses of PL was stained with 10 μM DCFH-DA and blocked by pre-incubated with 3 mM NAC for 2 h before exposure to PL. Intracellular ROS generation was measured by flow cytometry **(C**, **D)** or fluorescence microscope **(E)**. **(F**, **G)** DCF-DA mean fluorescence density was quantified using ImageJ. Data are expressed as the mean ± SD of three independent experiments. Bars = SD. *P < 0.05, **P < 0.01, ***P < 0.005.

P-gp is one of the pumps that can transport chemotherapeutic drugs from inside of tumor cells into outside, for which Rh-123 is a well-established substrate. Thus, activity of the P-gp drug pump can be evaluated by the degree of intracellular Rh-123 accumulation (Shi et al., 2008). Flow cytometry results indicated that 5 and 10 μM PL induced the accumulation of Rh-123 by 1.08-fold and 1.17-fold over control levels, respectively (**Figures 2C, D**). Moreover, the Rh-123’s density of PL treated A549/Cis cells is higher than that of A549 cells without treatment. These results indicated PL inhibited the cellular efflux pump activity of P-gp to increase the intracellular accumulation of anticancer drugs like cisplatin and 5 µM PL suppressed the protein expression of P-gp in A549/Cis cells (**Figures 3A, B**). Taken together, we suggested PL inhibited drug efflux in A549/Cis cells by suppressing the function and expression of P-gp.

### Phosphorylation of Akt Is the Key Target in the Chemotherapy Resistance Reversal Effect of PL in A549/Cis Cells

The PI3K/Akt/mTOR signaling pathway is one of the most important and attractive targets in anticancer therapy (Wang et al., 2017) and the Akt has a crucial regulatory role in lung tumorigenesis (Hollander et al., 2011). We next investigated its effects on the Akt signaling network. Treatment with 5 μM PL significantly altered the Akt signaling network. Phosphorylation of PTEN at Ser-380 was significantly increased, thereby inducing dephosphorylation of Akt at Ser-473 after PL treatment of A549/Cis cells (Reneer and Marti, 2012)(**Figures 3A, B**). As two of the best-established downstream targets of Akt (Manning and Toker, 2017), the phosphorylation levels of mTOR at Ser-2448 and FoxO3a at Ser-318/321 were evaluated (**Figures 3A, B**) and found to be significantly suppressed after PL treatment of A549/Cis cells. The dephosphorylation of FoxO3a caused by inhibition of Akt phosphorylation can lead to the transcriptional activation of Keap1, which in turn promotes drug efflux *via* inhibition of the NRF2/P-gp signaling pathway (Guan et al., 2016). The reduced expression of NRF2 and P-gp was also observed after PL treatment (**Figures 3A, B**). These results suggest that PL might promote drug efflux through the Akt/FoxO3/NRF2/P-gp signaling pathway. Akt suppresses apoptosis by phosphorylating its substrate BAD at Ser-75 and Ser-99 (Pandey et al., 2018). To further characterize the mechanisms underlying PL-induced apoptosis, Ser-75 and Ser-99 phosphorylation levels on BAD were examined (**Figures 3A, B**) and found to be significantly decreased after PL treatment in A549/Cis cells. Because p53, an important pro-apoptosis gene, is also degraded by Akt-mediated mouse double minute 2 homolog (MDM2) phosphorylation (Kawase et al., 2009). And p53’s expression was also measured and found to be significantly upregulated after PL treatment in A549/Cis cells (**Figures 3A, B**). In addition, expression of the anti-apoptotic protein Bcl-xL was significantly suppressed after PL treatment (**Figures 3A, B**). Either BAD or Bcl-xL is member of Bcl-2 family and regulate the first step for mitochondrial apoptosis (Xiong et al., 2014). These results demonstrated that PL treatment induced mitochondrial apoptosis in A549/Cis cells *via* dephosphorylation of Akt.

### Increased ROS Levels as a Result of PL Treatment Are Responsible for Akt Dephosphorylation

**Figure 4:**
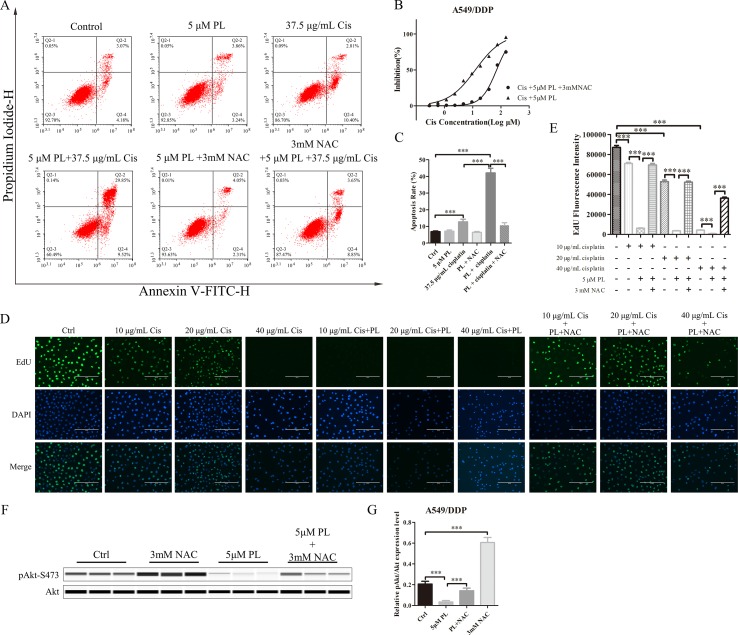
NAC can block the resistance reversal effect of PL in A549/Cis cells. **(A)** NAC antagonized the effect of PL combined with cisplatin on inducing apoptosis in A549/Cis cells. A549/Cis cells were treated with DMSO, 5 μM PL, 37.5 μg/ml cisplatin, 5 μM PL and 37.5 μg/ml cisplatin combined, 5 μM PL and 3mM NAC combined or 5 μM PL plus 37.5 μg/ml cisplatin, and 3mM NAC combined for 24 h. **(B)** the percentage of cell apoptosis was determined by Annexin–V/PI staining and flow cytometry in three independent experiments, and graphed by GraphPad Prism 5. **(C)** A549/Cis cells were treated with PL plus cisplatin alone or in combination with NAC at 24 h and cell viability was measured by an MTT assay. **(D)** A549/Cis cells were treated with increased dose of cisplatin alone, combined with PL or combined with PL and NAC, and the cell proliferation was measured by EdU assay and, **(E)** the fluorescence density was quantified using ImageJ. **(F)** A549/Cis cells were treated with DMSO, 3mM NAC, 5 μM PL alone, or in combination with NAC for 24 h. The effects on Akt and Akt (Ser-473) protein expression were evaluated by western blot analysis. **(G)** relative protein expression levels were quantified using ImageJ and normalized to Akt. Data are expressed as the mean ± SD of three independent experiments. Bars = SD. ***P < 0.005.

To investigate whether PL induces dephosphorylation of Akt *via* the ROS pathway, we measured the levels of intracellular ROS following treatment of cells with increasing concentrations of PL alone or combined with the well-established antioxidant NAC. ROS production increased in PL-treated cells in a dose-dependent manner and was blocked by the addition of NAC (**Figures 3C–G**). Furthermore, NAC completely reversed the chemosensibilization effect of PL in A549/Cis cells (**Figure 4C**). Moreover, the EdU assay showed that NAC could, against the inhibitory effect of Cis combined with PL treatmentin A549/Cis cell proliferation, even with a high dose of Cis (40 μg/ml; **Figures 4D, E**). We next examined the blocking effect of NAC in PL-induced cell apoptosis. NAC treatment had little effect on inducing cell apoptosis. By contrast, NAC completely blocked the apoptosis-induction effect of Cis/PL treatment in A549/Cis cells (**Figures 4A, B**). NAC also reversed the inhibition of Akt phosphorylation (Ser-473) by PL treatment (**Figures 4F, G**). These data showed that the chemotherapy reistance reversal effect of PL in A549/Cis cells depended on Akt phosphorylation *via* direct stimulation of ROS.

### PL Increases the Therapeutic Effects of Cis *In Vivo* Through Dephosphorylation of Akt

**Figure 5:**
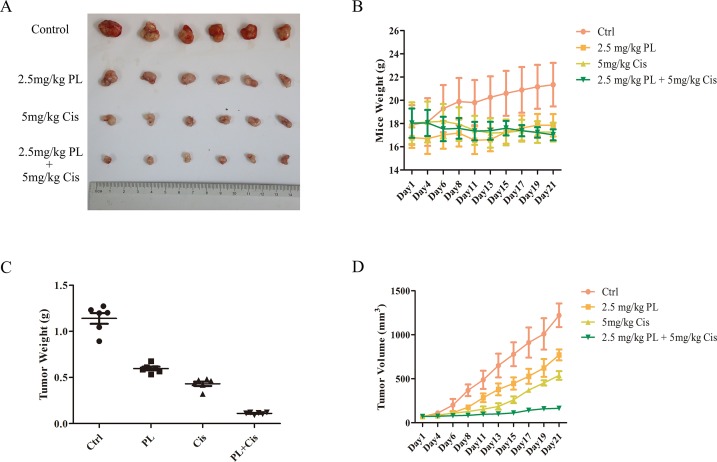
Piperlongumine (PL) and cisplatin inhibit *in vivo* tumor growth in cooperative manner. Antitumor effects of PL and cisplatin in a tumor xenograft mouse model. Nude mice were injected with 2 × 10^6^ A549/Cis cells in both flanks. Treatment with vehicle, PL, cisplatin, or the combination of PL and cisplatin began once the implanted tumor cells formed palpable nodules. Each group included six mice. Combined treatment inhibits tumor volume **(A**, **B)** and tumor weight **(C)** in nude mice, as well as the body weight of mice in cisplatin and combined treatment group is slightly decreased **(D)**. Bars = SD. *P < 0.05, ***P < 0.005.

**Figure 6:**
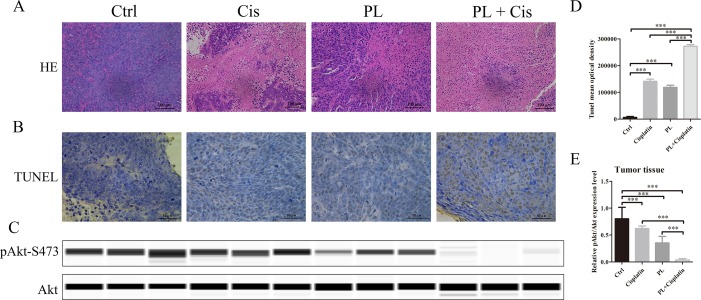
Piperlongumine (PL) combined with cisplatin treatment induces apoptosis through dephosphorylation of Akt *in vivo*. **(A)** The antitumor effect was evaluated by H&E staining. **(B)** *In vivo* apoptosis induced by drugs was measured by the TUNEL assay, **(C)** and quantified with mean optical density of each group using ImageJ. **(D)** Western blot analysis of Akt and Akt (Ser-473) proteins obtained from each group. **(E)** Relative protein expression levels were quantified using ImageJ and normalized to Akt. Bars = SD. ***P < 0.005.

To evaluate the combined effects of PL and Cis *in vivo*, BALB/c athymic nude mice bearing A549/Cis tumor xenografts were employed to investigate the therapeutic effects of Cis combined with PL. After 21 days, treatment with Cis and PL alone both inhibited tumor growth. However, tumor volume (**Figures 5A, B**) and weight (**Figure 5C**) were more effectively inhibited by the combined treatment of Cis and PL. Loss of weight was occurred either in mice with Cis treated alone or combined with PL, but there was little effect on the body weight of mice with PL treated alone (**Figure 5D**). Histopathological examination of heart, liver, lung, spleen, and renal from mice did not reveal any significant difference between the control and PL-treated groups, while cisplatin treatment caused degeneration but without any obvious necrosis of hepatocytes as well as the infiltration of inflammatory cells or proliferation of fibrous tissue in hepatic lobules and portal canal areas. Also, changes in kidney structure with turbid swelling of nephric convoluted epithelial cells in cisplatin treated mice were observed (data not shown). Histopathological examination also suggested that the combined treatment of Cis and PL markedly reduced the mitosis of tumor cells and induced the necrosis of tumor tissue compared with the control (**Figure 6A**). In situ apoptosis assays showed that apoptotic cells were more frequently seen in tumors treated with PL/Cis than in those treated with vehicle (**Figures 6B, C**). Western blot analyses of tumor tissues showed that Akt phosphorylation (Ser-473) was decreased in both the PL and PL/Cis treatment groups compared with the vehicle group; however, it was suppressed to a greater extent in the PL/Cis group (**Figures 6D, E**). These results demonstrated that PL promoted the therapeutic effects of Cis through dephosphorylation of Akt.

## Discussion

Chemotherapy plays an essential role in cancer treatment and is often employed after surgery as an adjuvant therapy in patients with NSCLC (Salazar et al., 2017). However, drug resistance develops in nearly all patients (O’grady et al., 2014). Therefore, identifying effective chemosensitizers and combining them with anticancer agents is a promising strategy for overcoming chemotherapy resistance (Robert and Jarry, 2003). Several studies had discovered some compounds as chemosensitizers *in vitro* including verapamil, cyclosporine, and tamoxifen. However, they have limited clinical applications due to their high toxicity and low activity (Robert and Jarry, 2003). PL can reverse chemotherapy resistance and regulated the expression of various genes in a variety of resistance cancer cells, while exhibiting low toxicity and high activity (Farrand et al., 2013; Gong et al., 2014; Kang and Yan, 2015; Wang et al., 2015), but the mechanisms are still unclear. Thus, a comprehensive understanding of the underlying mechanisms of PL is critical for the development and improvement of novel treatment strategies for patients with chemotherapy resistance.

In this study, cytotoxicity assays indicated that A549/Cis cells had resistance to cisplatin, PL cytotoxicity is concentration-dependent and there is no difference between A549 and A549/Cis cells. To investigate the reversal effect of PL on A549/Cis cells, PL was administered to cells at the weakly cytotoxic concentration of 5 μM and enhanced the cytotoxicity of Cis by more than 5-fold. Thus, PL partially reversed the drug resistance of A549/Cis cells. The reduced apoptosis rate of resistance cancer cells is one major cause of chemotherapy resistance, which involves the alterations of genes and proteins that control apoptosis (Baritaki et al., 2007). Apoptosis studies demonstrated the higher apoptosis rate by PL plus cisplatin combination compared with individual drugs *in vitro* and *in vivo*. Transporting to extracellular of chemotherapeutics is another major cause of chemotherapy resistance (Xu et al., 2006). A well-accepted hypothesis is that intracellular levels of chemotherapeutics are reduced below lethal thresholds by active extrusion, through the regulation of ATP-dependent pumps such as P-gp. Accumulation assays using Rh-123 demonstrated the blocking effects of PL on the cellular efflux pump activity of P-gp, thereby inducing the intracellular accumulation of Cis. Thus, PL reversed the resistance to Cis of A549/Cis cells by promoting apoptosis and reducing Cis efflux.

**Figure 7:**
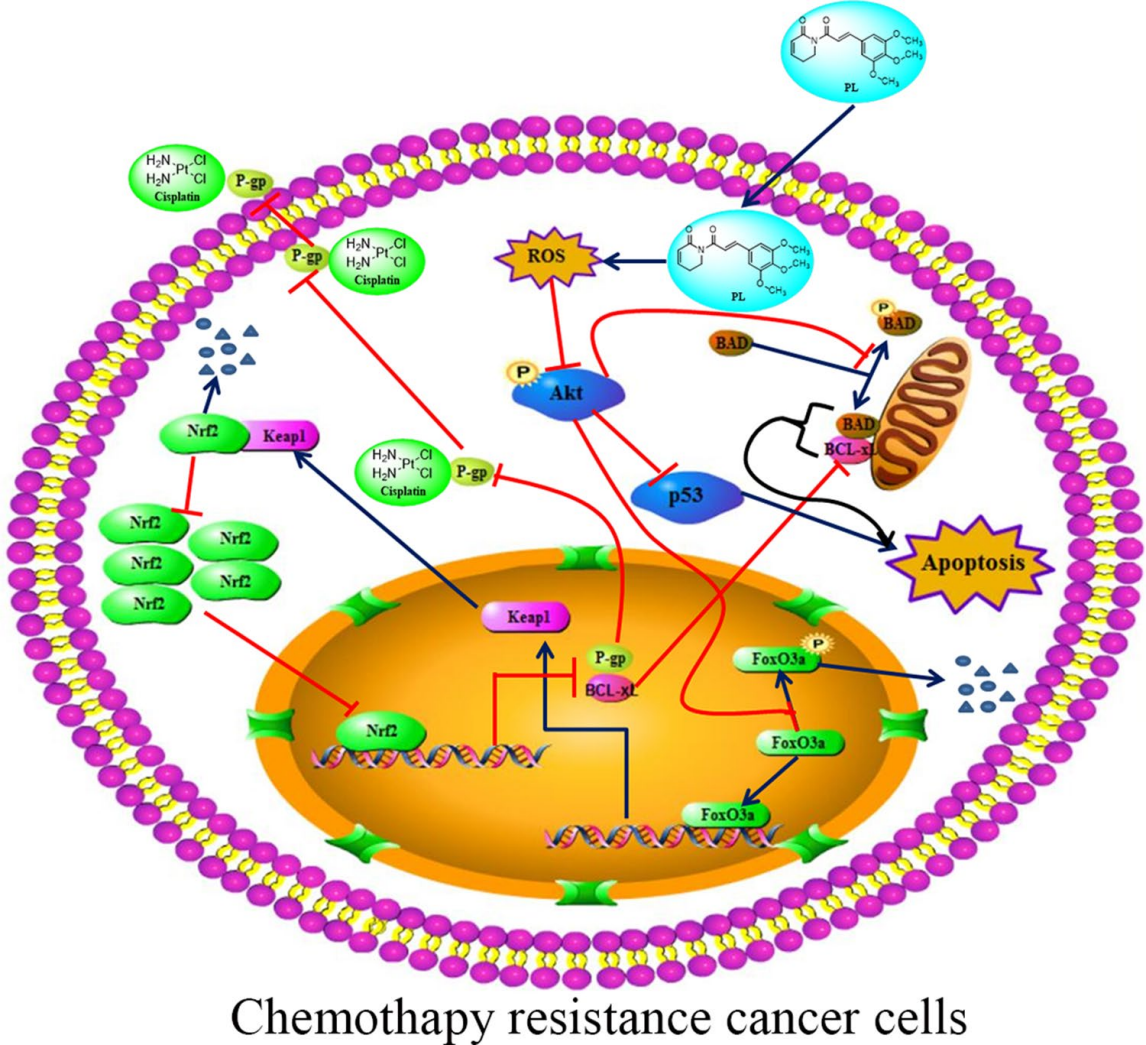
Proposed signaling pathway for how Piperlongumine (PL) reverses the resistance of cisplatin in NSCLC. PL induces intracellular ROS accumulation, which in turn inhibits the phosphorylation of Akt and its network leading to induced apoptosis and reduced cisplatin efflux.

The Akt signaling pathway plays a crucial regulatory role in cell proliferation, survival, and the sensitivity of cancer cells to Cis (Hayakawa et al., 2000; Hay, 2005). Recent studies have demonstrated an anti-tumor role for ROS, which occurs through several distinct mechanisms (Sabharwal and Schumacker, 2014). ROS also functions as an important physiological regulator of Akt signaling (Zhao et al., 2017; Moldogazieva et al., 2018). In addition to its positive modulating effects on Akt signaling, ROS exerts direct effects on Akt under conditions of oxidative stress (Hussain et al., 2011; Shearn et al., 2011). Our data showed that PL acts as a ROS inducer to directly promote dephosphorylation of Akt at Ser-473. As shown in **Figure 7**, dephosphorylation of Akt suppressed phosphorylation of Foxo3, which may stabilize and promote the function of Foxo3, subsequently inducing Keap1 expression, inhibiting the transcriptional function of NRF2, and ultimately reducing P-gp expression. PL treatment affected the Akt/Foxo3/NRF2/P-gp, signaling pathway to inhibit Cis efflux in A549/Cis cells. However, dephosphoryation of Akt reduced BAD phosphorylation, causing downregulation of Bcl-xL by NRF2 inactivation, together promoting formation of heterodimers with BAD and Bcl-xL to induce apoptosis. In addition, Akt dephosphorylation was also responsible for the upregulated p53 expression observed in A549/Cis cells with PL treatment. Thus, PL treatment promoted A549/Cis cell apoptosis *via* an Akt-mediated network including BAD, Bcl-xL, and p53. Administration of NAC completely reversed all of the chemotherapy resistance reversal effects of PL in A549/Cis cells and induction of Akt phosphorylation occurred in cells treated with both NAC and PL, confirming our hypothesis that PL inhibition of Akt signaling was mediated by ROS.

In conclusion, this study confirmed the reversal effects of PL on MDR in A549/Cis cells and to the best of our knowledge, demonstrates for the first time that PL induces ROS accumulation and directly reduces Akt phosphorylation. This, in turn, suppresses Akt signaling, consequently rendering A549/Cis cells sensitive to Cis by promoting apoptosis and inhibiting Cis efflux. Therefore, the combination of PL with cytotoxic drugs may serve as a promising therapeutic strategy for NSCLC patients with chemotherapy resistance.

## Data Availability Statement

All datasets generated for this study are included in the manuscript /supplementary files.

## Ethics Statement

This study was carried out in accordance with the recommendations of Wannan Medical College’s Policy on the Care and Use of Laboratory Animals. The protocol was approved by the Wannan Medical College Animal Policy and Welfare Committee.

## Author Contributions

X-PL and CZ designed and provided guidance for the study. L-JH and CZ performed the experiments, analyzed the data, and wrote the manuscript. Y-BZ, Q-ZF, D-DM, S-PZ, and W-YZ participated in some of the experiments.

## Funding

The present study was supported by the National Natural Science Foundation of China (grant no. 81272485), the Natural Science Foundation of Anhui Province (grant nos. 1808085QH262 and 1708085MH202), the University Natural Science Research Project of Anhui Province (grant no. KJ2018A0259), Specialized Research Fund for the Doctoral Program of Wannan Medical College (grant no. rcqd201607), and Key Research Cultivation Foundation of Wannan Medical College (grant no.WK2017Z03).

## Conflict of Interest

The authors declare that the research was conducted in the absence of any commercial or financial relationships that could be construed as a potential conflict of interest.
